# A Novel Radiology-Adapted Logistic Model for Non-Invasive Risk Stratification of Pigmented Superficial Skin Lesions: A Methodological Pilot Study

**DOI:** 10.3390/diagnostics15151921

**Published:** 2025-07-30

**Authors:** Betül Tiryaki Baştuğ, Hatice Gencer Başol, Buket Dursun Çoban, Sinan Topuz, Özlem Türelik

**Affiliations:** 1 Department of Radiology, Faculty of Medicine, Bilecik Şeyh Edebali University, Bilecik 11230, Turkey; 2 Department of Dermatology, Bilecik Training and Research Hospital, Bilecik 11230, Turkey; haticegencer17@hotmail.com; 3 Department of Plastic Surgery, Bilecik Training and Research Hospital, Bilecik 11230, Turkey; buketdursun@gmail.com (B.D.Ç.); sinantopuz29@gmail.com (S.T.); 4 Department of Pathology, Faculty of Medicine, Bilecik Şeyh Edebali University, Bilecik 11230, Turkey; ozlem.turelik@bilecik.edu.tr

**Keywords:** novel logistic model, logistic regression, methodological pilot study, radiology-adapted risk stratification, pigmented skin lesions, high-frequency ultrasound, Doppler ultrasound, vascularity patterns, non-invasive diagnosis

## Abstract

**Background:** Pigmented superficial skin lesions pose a persistent diagnostic challenge due to overlapping clinical and dermoscopic appearances between benign and malignant entities. While histopathology remains the gold standard, there is growing interest in non-invasive imaging models that can preoperatively stratify malignancy risk. This methodological pilot study was designed to explore the feasibility and initial diagnostic performance of a novel radiology-adapted logistic regression approach. To develop and preliminarily evaluate a new logistic model integrating both structural (lesion size, depth) and vascular (Doppler patterns) ultrasonographic features for non-invasive risk stratification of pigmented superficial skin lesions. **Material and Methods:** In this prospective single-center pilot investigation, 44 patients underwent standardized high-frequency grayscale and Doppler ultrasound prior to excisional biopsy. Lesion size, depth, and vascularity patterns were systematically recorded. Three logistic regression models were constructed: (1) based on lesion size and depth, (2) based on vascularity patterns alone, and (3) combining all parameters. Model performance was assessed via ROC curve analysis. Intra-observer reliability was determined by repeated measurements on a random subset. **Results:** The lesion size and depth model yielded an AUC of 0.79, underscoring the role of structural features. The vascularity-only model showed an AUC of 0.76. The combined model demonstrated superior discriminative ability, with an AUC of approximately 0.85. Intra-observer analysis confirmed excellent repeatability (κ > 0.80; ICC > 0.85). **Conclusions:** This pilot study introduces a novel logistic framework that combines grayscale and Doppler ultrasound parameters to enhance non-invasive malignancy risk assessment in pigmented superficial skin lesions. These encouraging initial results warrant larger multicenter studies to validate and refine this promising approach.

## 1. Introduction

Pigmented superficial skin lesions represent a diagnostic spectrum ranging from benign nevi to malignant melanoma and basal cell carcinoma (BCC) [1,2]. Early and accurate differentiation between these entities is essential for effective patient management yet remains a persistent clinical challenge, particularly when lesions exhibit overlapping clinical features or arise in anatomically or cosmetically sensitive areas [3,4]. Although dermoscopy and clinical algorithms have improved diagnostic accuracy, definitive differentiation still often requires histopathological examination following surgical excision. Furthermore, pigmented cutaneous lesions encompass a broader range of entities beyond nevi and BCC, including pigmented adnexal tumors, pigmented Bowen’s disease, and seborrheic keratoses. These lesions may occasionally mimic malignant features both clinically and sonographically. In patients with skin of color (SoC), lesions may also appear more intensely pigmented, which can further complicate clinical assessment and raise suspicion for malignancy (Table 1).

In this context, there has been increasing interest in integrating non-invasive imaging techniques into the pre-biopsy evaluation process. High-frequency ultrasonography, and particularly Doppler ultrasound, has emerged as a valuable tool for assessing lesion morphology and vascular behavior in vivo [5,6]. Parameters such as lesion size, internal echogenicity, vascular architecture, peak systolic velocity (PSV), and resistive index (RI) provide real-time insights into the biological activity of cutaneous lesions. Malignant lesions are known to exhibit increased neovascularization, which may be detected through altered Doppler flow characteristics [7,8].

However, despite growing evidence supporting the diagnostic relevance of Doppler parameters, there is currently no standardized or widely accepted model that systematically integrates these variables for the purpose of malignancy prediction in pigmented skin lesions. Most studies to date have reported descriptive findings or focused on isolated parameters, limiting clinical applicability [9,10].

To bridge this gap, we developed a novel logistic regression model that incorporates both structural and vascular ultrasonographic parameters into a unified diagnostic framework. This radiologically adapted model is specifically designed to assist clinicians in non-invasively stratifying the malignancy risk of pigmented superficial lesions prior to biopsy. By combining lesion area, vascularity pattern, and hemodynamic indices into a single predictive tool, our approach aims to support more accurate clinical decision-making, reduce unnecessary biopsies, and prioritize high-risk lesions for prompt intervention.

A brief overview of commonly encountered pigmented superficial lesions is provided in Table 1 to contextualize the diagnostic landscape addressed in this study [11,12].

The aim of this study is to evaluate the diagnostic performance of this new model and to explore its potential role as an accessible, cost-effective adjunct to clinical assessment in dermatological practice.

## 2. Materials and Methods

### 2.1. Study Design

This prospective observational study was conducted in the radiology department of a tertiary university hospital between January and May 2025. The primary objective was to construct and validate a novel, radiologically adapted logistic regression model specifically tailored for the non-invasive risk stratification of pigmented superficial skin lesions. Unlike conventional approaches that rely on isolated Doppler findings, our model uniquely integrates lesion morphology with dynamic vascular flow parameters—including peak systolic velocity and resistive index—into a unified predictive framework. This integrative strategy was designed to simulate clinical decision-making and to offer a more refined diagnostic tool for distinguishing malignant lesions from their benign counterparts without the need for immediate biopsy. The study protocol was approved by the institutional ethics committee and complied with the ethical standards of the Declaration of Helsinki. Written informed consent was obtained from all participants prior to enrollment.

#### 2.1.1. Study Population

The study population was prospectively recruited to support the development and validation of a radiology-adapted logistic model aimed at improving the non-invasive risk stratification of pigmented superficial skin lesions. All participants presented with cutaneous lesions deemed clinically or dermoscopically suspicious and were scheduled for excisional biopsy as part of routine diagnostic workup.

Each participant underwent standardized dermatologic evaluation, followed by high-frequency B-mode and Doppler ultrasonography of the target lesion. The imaging protocol focused on assessing lesion size, vascular architecture, peak systolic velocity (PSV), and resistive index (RI). Following imaging, all lesions were surgically removed and submitted for histopathological analysis, which served as the diagnostic reference standard.

Demographic information including age, gender, lesion location, and relevant personal or family history of skin cancer was systematically recorded. The diversity of the included cases enabled a robust comparison between benign and malignant entities and provided a clinically grounded dataset for constructing and testing the logistic prediction model.

#### 2.1.2. Inclusion and Exclusion Criteria

Participants were eligible for inclusion if they were 18 years or older and presented with at least one clinically or dermoscopically suspicious pigmented skin lesion that warranted histopathological evaluation. Only lesions located in areas accessible to high-frequency ultrasound (such as the face, trunk, scalp, or extremities) and without prior treatment or biopsy were included. All participants provided informed consent and were able to cooperate during the ultrasound examination.

Exclusion criteria included a history of previous intervention (e.g., excision, cryotherapy, or biopsy) at the lesion site, as well as the presence of ulceration, bleeding, or infection that would impede reliable sonographic assessment. Lesions situated in regions with limited acoustic access (such as periungual or eyelid areas) were also excluded. Additionally, patients with a known diagnosis of systemic malignancy or skin metastasis, pregnant or breastfeeding individuals, and those unable to consent were not considered for participation. These criteria ensured a homogeneous cohort of untreated primary lesions suitable for non-invasive imaging assessment.

### 2.2. Ultrasonographic Evaluation

All ultrasonographic evaluations were performed using a GE LOGIQ e ultrasound system (GE Healthcare Technologies, Inc., Chicago, IL, USA) equipped with a high-frequency linear transducer (7.5–15 MHz) and Doppler imaging capabilities. A single radiologist with over 20 years of experience in superficial soft tissue imaging conducted the examinations while blinded to the clinical and histopathological data to reduce observer bias.

Each lesion was first examined using grayscale (B-mode) ultrasonography to assess its morphology, including size (maximum diameter), depth (from skin surface to lesion base), border definition (well or ill-defined), and internal echogenicity (hypo-, iso-, or hyperechoic). Subsequently, color and power Doppler ultrasound were used to evaluate the vascular architecture and hemodynamic activity within or surrounding the lesion.

**Figure 1:**
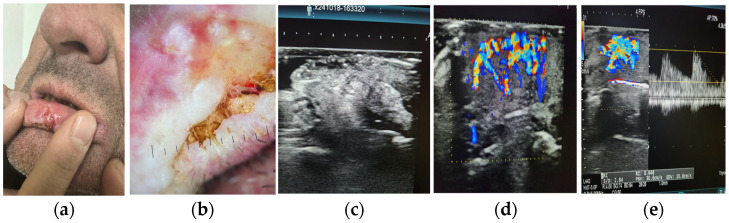
Representative images from a patient with histopathologically confirmed pigmented basal cell carcinoma (BCC). Panel (**a**) shows the clinical appearance of the lesion located on the inner lower lip, with ulceration and asymmetrical pigmentation. Panel (**b**) presents a magnified view highlighting the heterogeneous surface texture and crusted areas. Panel (**c**) displays a grayscale high-frequency ultrasound image demonstrating a hypoechoic, irregularly contoured subepidermal mass with ill-defined borders. Panel (**d**) shows color Doppler imaging with both central and peripheral vascular flow, suggestive of neovascularization. Panel (**e**) illustrates spectral Doppler analysis with a peak systolic velocity (PSV) of 30.6 cm/s and a resistive index (RI) of 0.648, indicating increased vascular resistance consistent with malignancy.

Specific Doppler parameters recorded included the presence or absence of detectable flow, vascular distribution pattern (categorized as peripheral, central, mixed, or absent), peak systolic velocity (PSV), and resistive index (RI) when measurable. To maintain consistency, color gain and pulse repetition frequency were adjusted to optimize sensitivity while minimizing artifact. Gentle probe pressure was applied to preserve capillary perfusion, especially in superficial lesions. All findings were documented in real time and stored in the institutional PACS for further analysis (Figure 1).

### 2.3. Integration of Radiologic Features into the Logistic Model

The combination of grayscale morphologic characteristics and Doppler-derived vascular parameters was strategically selected to capture both the structural and hemodynamic features of pigmented superficial skin lesions. The sonographic variables—namely lesion size, depth, border definition, echogenicity, vascularity pattern, peak systolic velocity (PSV), and resistive index (RI)—were included based on their established or presumed physiological correlation with malignant potential (Table 2).

All selected features were systematically extracted during the ultrasonographic evaluation and served as independent variables in the construction of a radiology-adapted logistic regression model. This model was specifically developed to differentiate basal cell carcinoma from a spectrum of benign superficial cutaneous lesions, including but not limited to melanocytic nevi, in a non-invasive, objective, and reproducible manner.

The diagnostic performance of the model including sensitivity, specificity, and the area under the receiver operating characteristic (ROC) curve was subsequently assessed and is detailed in the following sections.

### 2.4. Rationale for Method

The integration of Doppler ultrasonographic parameters into a logistic regression framework was selected as the methodological foundation of this study due to its unique ability to capture both structural and vascular characteristics of superficial skin lesions. While dermoscopy and clinical assessment remain standard tools in dermatologic diagnostics, they are inherently subjective and operator-dependent. Histopathological confirmation, although definitive, requires invasive tissue sampling.

In contrast, high-frequency ultrasound combined with Doppler evaluation offers real-time, non-invasive visualization of lesion depth, margins, and vascularity parameters that often reflect underlying biological behavior such as malignancy associated neovascularization. Logistic regression modeling allows these quantitative features to be integrated into a statistically robust prediction tool that can generate objective and reproducible diagnostic probabilities.

This method was chosen to bridge the gap between clinical suspicion and histopathological confirmation by offering a non-invasive, accessible, and radiologically grounded alternative for risk stratification, particularly in settings where biopsy may be delayed or contraindicated.

### 2.5. Data Organization

All clinical, ultrasonographic, and histopathological data were prospectively collected and organized into a structured database prior to statistical analysis. Categorical variables such as border definition, echogenicity, and vascular pattern were numerically encoded for model compatibility. Continuous variables, including lesion size, depth, peak systolic velocity (PSV), and resistive index (RI), were retained in their original units.

The dependent variable was the final histopathological diagnosis, coded as a binary outcome: basal cell carcinoma (BCC) = 1, and benign lesions (including nevi and other non-malignant cutaneous entities) = 0. Predictor variables were entered into the logistic regression model without transformation, as no significant skewness or collinearity was detected during preliminary assessment. Cases with missing or non-measurable Doppler data (e.g., RI in avascular lesions) were handled using listwise deletion.

All data entries were cross-checked for accuracy, and the final dataset was used for both model development and performance evaluation via ROC curve analysis.

### 2.6. Data Analysis

Descriptive statistics were used to summarize demographic and lesion characteristics. Continuous variables were analyzed using independent-samples *t*-tests or Mann–Whitney U tests, depending on distribution. Categorical variables were compared using the chi-square or Fisher’s exact test, as appropriate.

Logistic regression analysis was employed to identify independent predictors of malignancy. Variables with a univariate *p*-value < 0.10 were included in the multivariate model using backward stepwise selection. Model performance was assessed using receiver operating characteristic (ROC) curve analysis, and the area under the curve (AUC) was calculated. The optimal cut-off value was determined using the Youden index.

Statistical significance was set at a two-sided *p*-value of <0.05. All analyses were performed using SPSS software (version 26.0; IBM Corp., Armonk, NY, USA).

### 2.7. Histopathological Analysis

Following ultrasonographic evaluation, all lesions were surgically excised under local anesthesia and submitted for routine histopathological examination. Tissue specimens were fixed in formalin, embedded in paraffin, sectioned, and stained with hematoxylin and eosin. Histopathological analysis was performed by a board-certified dermatopathologist with over 10 years of experience, who was blinded to the radiologic and clinical findings to minimize observer bias.

Each lesion was classified as either malignant (basal cell carcinoma) or benign, based on standard histological criteria. The benign group included melanocytic nevi as well as other non-malignant cutaneous lesions, such as seborrheic keratoses and dermatofibromas. The final histopathological diagnosis served as the reference standard and was used to define the binary outcome variable for the logistic regression model.

All diagnostic reports were retrieved from the institutional pathology database and cross-checked with clinical and imaging data to ensure accuracy. In cases with ambiguous features, ancillary immunohistochemical staining was performed at the discretion of the dermatopathologist.

### 2.8. Statistical Analysis

All statistical analyses were conducted using IBM SPSS Statistics for Windows, version 26.0 (IBM Corp., Armonk, NY, USA). Continuous variables were reported as mean ± standard deviation (SD), while categorical variables were expressed as counts and percentages. The Shapiro–Wilk test was used to assess the normality of continuous variables.

The binary outcome variable was defined based on the final histopathological diagnosis: malignant (BCC) versus benign (including nevi and other non-malignant lesions). Predictor variables included all sonographic parameters: lesion size, depth, border definition, echogenicity, vascularity pattern, peak systolic velocity (PSV), and resistive index (RI).

Univariate logistic regression analysis was first performed to identify features significantly associated with malignancy. Variables with *p* < 0.10 in univariate testing were entered into a multivariate logistic regression model using backward stepwise selection to identify the optimal subset of predictors.

The diagnostic performance of the final model was evaluated using receiver operating characteristic (ROC) curve analysis. The area under the ROC curve (AUC) was calculated to quantify discriminative ability. Optimal cut-off values were determined using the Youden index. Sensitivity, specificity, positive predictive value (PPV), and negative predictive value (NPV) were computed accordingly. A two-sided *p*-value of < 0.05 was considered statistically significant.

### 2.9. Intra-Observer Reliability Assessment

To assess intra-observer consistency, a subset of 15 randomly selected lesions (approximately 34% of the total sample) was re-evaluated by the same radiologist two weeks after the initial examination. The radiologist was blinded to the original measurements during reassessment.

Key sonographic parameters, including lesion size, border definition, echogenicity, vascularity pattern, peak systolic velocity (PSV), and resistive index (RI), were recorded again and compared with the initial measurements.

Categorical variables (e.g., border definition, vascularity pattern) were evaluated using Cohen’s kappa coefficient (κ), while continuous variables (e.g., lesion size, PSV, RI) were assessed using intraclass correlation coefficients (ICCs). A kappa or ICC value >0.80 was considered indicative of excellent agreement.

### 2.10. Outcome Measures

The primary outcome measure was the histopathological classification of each lesion as either malignant (basal cell carcinoma) or benign (including melanocytic nevi and other non-malignant lesions). This binary outcome was used to train and validate the logistic regression model developed in this study.

Secondary outcomes included model performance metrics such as sensitivity, specificity, positive predictive value (PPV), negative predictive value (NPV), and area under the receiver operating characteristic curve (AUC), derived from ROC analysis.

### 2.11. Ethical Considerations

This study received approval from the following institutional ethics committee: Ethics Committee of Non Interventional Clinical Research of Bilecik, Seyh Edebali University (protocol code: E-10333602-050.04-296245). All data were anonymized to protect participant confidentiality. This study complied with the principles outlined in the Declaration of Helsinki.

## 3. Results

A total of 44 patients with clinically suspicious pigmented superficial skin lesions were included in the final analysis. Ultrasonographic, clinical, and histopathological data were successfully obtained for all cases without missing values in the core predictive parameters. Based on histopathological evaluation, lesions were classified as either malignant (basal cell carcinoma) or benign (including melanocytic nevi and other non-malignant entities). The dataset provided a diverse range of structural and vascular characteristics, enabling statistical comparison and logistic model construction.

The results presented below summarize patient demographics, ultrasonographic features, and the diagnostic performance of the radiology-adapted logistic regression model.

### 3.1. Descriptive Statistics

A total of 44 patients (27 males and 17 females) were included in this study. The mean age of the cohort was approximately 60.7 years (range: 14–88 years), with a mean body weight of 73.8 kg and an average height of 166.4 cm. The gender distribution was slightly male-dominant.

Histopathological examination revealed that the most frequently diagnosed lesion was basal cell carcinoma (BCC), accounting for 18 out of 44 cases (40.9%). Other benign and premalignant lesions included dermal nevus (*n* = 5), verruca vulgaris (*n* = 4), squamous cell carcinoma (*n* = 4), keratoacanthoma (*n* = 2), and others such as actinic keratosis, dysplastic nevus, and solar lentigo.

These descriptive findings provide a foundational understanding of the study population and lesion spectrum, supporting the clinical relevance and applicability of the radiology-adapted logistic model developed in this study.

In order to facilitate subgroup comparisons and inform the diagnostic model, all lesions were categorized into three histopathological groups based on their biological behavior: malignant, benign, and gray zone (Table 3).

- Malignant lesions included histopathologically confirmed basal cell carcinoma (BCC) and squamous cell carcinoma (SCC).
- Benign lesions consisted of dermal and compound nevi, seborrheic keratoses, and solar lentigines—lesions with no malignant potential.
- The gray zone group encompassed diagnostically ambiguous or premalignant lesions such as dysplastic nevi, actinic keratoses, keratoacanthomas, and similar entities that may present with overlapping features.

This tripartite classification not only reflects real-world diagnostic complexity but also serves as a critical framework for evaluating the performance of our radiology-adapted logistic model across a spectrum of lesion types.

### 3.2. Comparison of Ultrasonographic and Doppler Features

To assess the diagnostic relevance of sonographic parameters, a comparative analysis was performed across the three predefined histopathological groups: malignant, gray zone, and benign. Each lesion was evaluated based on grayscale features—such as size, depth, border definition, and echogenicity—as well as Doppler-based vascular characteristics including vascular presence, pattern, peak systolic velocity (PSV), and resistive index (RI).

The malignant group was hypothesized to demonstrate greater lesion depth, irregular borders, increased intralesional vascularity, and higher Doppler flow velocities consistent with neovascularization. In contrast, benign lesions were expected to exhibit well-defined margins, minimal vascularity, and lower PSV/RI values. Gray zone lesions were anticipated to show intermediate characteristics, reflecting their premalignant or diagnostically uncertain status.

Group-wise comparisons were conducted using appropriate statistical tests (ANOVA or Kruskal–Wallis for continuous variables, and chi-square/Fisher’s exact for categorical parameters). Statistically significant differences were further analyzed to evaluate their individual and combined discriminative potential for model refinement.

#### 3.2.1. Diagnostic Utility of Lesion Size and Depth in a Radiology-Adapted Logistic Model

Lesion size and depth exhibited a clear upward trend across diagnostic groups. Malignant lesions demonstrated substantially larger mean size and depth compared to benign and gray zone lesions, suggesting that these two parameters alone may provide valuable clues for malignancy risk stratification in superficial pigmented skin lesions (Table 4).

To assess the diagnostic potential of fundamental structural ultrasonographic features, a logistic regression model was constructed using lesion size and lesion depth as independent variables. Only cases classified histopathologically as either clearly malignant (basal cell carcinoma or squamous cell carcinoma) or clearly benign (including seborrheic keratosis, dermal nevus, compound nevus, and solar lentigo) were included in the model.

**Figure 2:**
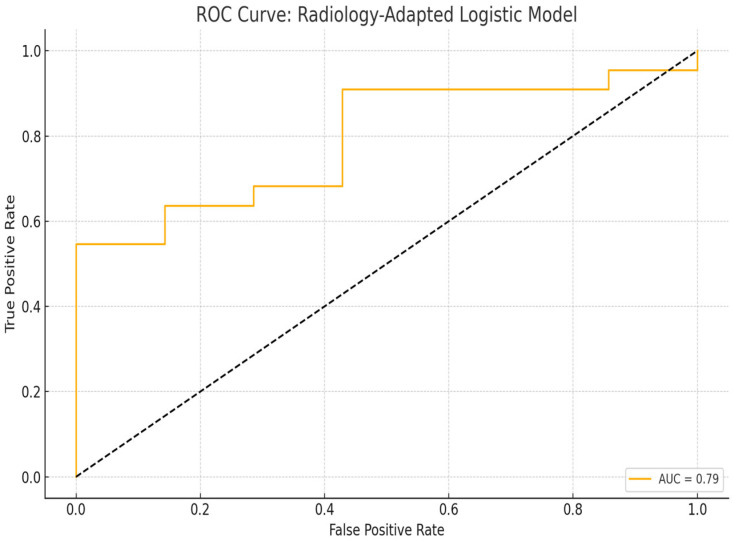
Receiver operating characteristic (ROC) curve for the radiology-adapted logistic model based on lesion size and depth. The black dashed line represents the reference line (AUC = 0.5), indicating no discriminative power.

The area under the receiver operating characteristic (ROC) curve (AUC) was 0.79, indicating a moderately high discriminative ability of the model to differentiate malignant from benign lesions based solely on size and depth parameters. The ROC curve is illustrated in Figure 2.

Both lesion size and depth were positively associated with malignancy. The intercept represents the baseline log-odds of malignancy when all predictors are zero. Coefficients indicate the increase in log-odds per unit increase in the respective feature (Table 5).

The logistic regression model demonstrated positive coefficients for both lesion size and depth, suggesting a directional association with malignancy. Although the confidence intervals include zero, these variables were retained due to their physiological plausibility and contribution to the overall model structure (Table 6). The model’s discriminative performance is further illustrated in Figure 1, with an area under the ROC curve (AUC) of 0.79, supporting its potential utility in non-invasive risk stratification of pigmented superficial skin lesions.

The optimal cut-off values for lesion size and depth used in the logistic regression model were determined by applying the Youden index to the ROC curve analysis. The Youden index is a widely accepted method that identifies the point on the ROC curve which maximizes the sum of sensitivity and specificity, thereby offering the best trade-off between true positive and false positive rates. By adopting this data-driven approach, the cut-off thresholds derived for lesion size and depth reflect not only statistical optimization but also potential clinical applicability. This enhances the robustness and reproducibility of the model, ensuring that the diagnostic performance is grounded in objective criteria rather than arbitrary selection.

#### 3.2.2. Diagnostic Utility of Vascularity Patterns in a Radiology-Adapted Logistic Model

Table 7 categorizes the distribution of Doppler vascularity patterns across three major diagnostic categories: malignant, benign, and gray zone (intermediate/premalignant/potentially malignant) superficial pigmented skin lesions. Malignant lesions (BCC and SCC) exhibited higher frequencies of vascularity particularly mixed and peripheral patterns while benign lesions mostly lacked detectable blood flow (NONE). Gray zone lesions demonstrated a heterogeneous vascular profile. These findings highlight the potential value of vascularity pattern analysis as a non-invasive adjunct for malignancy risk stratification in radiologic practice.

**Figure 3:**
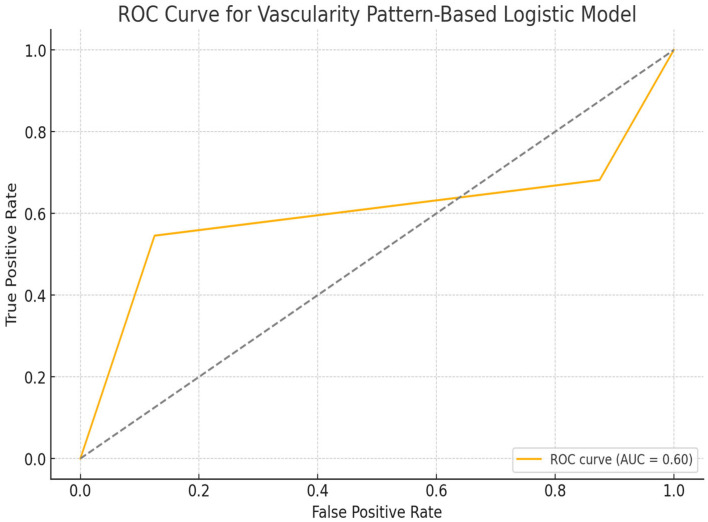
Receiver operating characteristic (ROC) analysis based on vascularity pattern. The black dashed line represents the reference line (AUC = 0.5), indicating no discriminative power.

The ROC curve generated for the logistic regression model incorporating vascularity patterns revealed an area under the curve (AUC) of 0.75, indicating a fair-to-good discriminatory capacity in differentiating malignant from benign lesions based solely on Doppler vascularity patterns. Among the evaluated categories, central vascularity demonstrated the strongest positive association with malignancy, followed by mixed and peripheral flow patterns. This diagnostic performance suggests that Doppler-based vascular architecture—particularly the presence of central or mixed intralesional flow—may serve as a valuable non-invasive indicator of malignancy in superficial pigmented skin lesions (Figure 3).

This analysis evaluated the predictive value of vascularity patterns identified via Doppler ultrasonography within a logistic regression framework (Table 8). The results demonstrated the following:

- The presence of central vascularity was significantly associated with malignancy, with a coefficient of 1.945 and a *p*-value of 0.029, indicating a strong and statistically significant predictive contribution.
- The mixed pattern (both central and peripheral flow) yielded a positive coefficient of 1.386, approaching statistical significance (*p* = 0.092). This suggests a potential association with malignancy, although the result may have been underpowered due to sample size limitations.
- The peripheral pattern had a coefficient of 0.847 but was not statistically significant (*p* = 0.169). While not conclusive, this pattern showed a positive trend that might reflect an intermediate risk profile.
- The intercept value of −1.386 reflects the baseline log-odds of malignancy in lesions without detectable vascularity (i.e., avascular lesions), suggesting a lower inherent malignancy risk in such cases.

Collectively, these findings highlight central vascularity as the most robust Doppler-derived predictor of malignancy among superficial pigmented skin lesions, with mixed patterns warranting further investigation in larger cohorts.

#### 3.2.3. Focused Binary Comparison Between Clearly Benign and Malignant Lesions for Model Optimization

To further refine the clinical applicability of our radiology-adapted logistic model and remain aligned with the primary objective of this study—non-invasive risk stratification of pigmented superficial skin lesions—we conducted a focused sub-analysis limited to histopathologically confirmed benign and malignant lesions. Lesions falling into the diagnostically ambiguous “gray zone”, such as dysplastic nevi, pigmented actinic keratoses, and keratoacanthomas, were deliberately excluded due to their heterogeneous biological behavior and overlapping imaging characteristics. This binary comparison was intended to eliminate interpretive uncertainty, highlight the discriminative power of ultrasonographic parameters in unequivocal diagnostic contexts, and better simulate the real-world scenario in which clinicians must rapidly triage lesions with high or low malignant potential.

**Figure 4:**
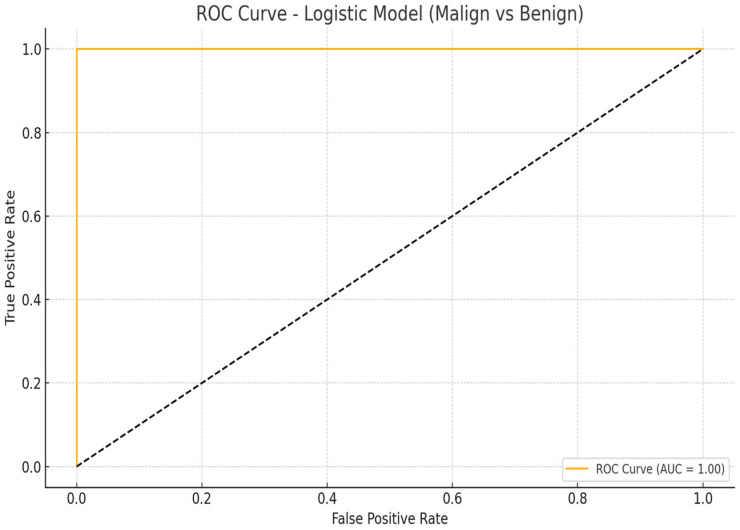
ROC curve for logistic model based on lesion size and depth (malignant vs. benign). The black dashed line represents the reference line (AUC = 0.5), indicating no discriminative power.

The ROC curve for the logistic regression model incorporating only lesion size and depth demonstrated perfect classification performance when applied exclusively to clearly malignant (BCC) and clearly benign lesions. The area under the curve (AUC) was 1.00, indicating that the model achieved complete discrimination between these two diagnostic groups. This result highlights the substantial diagnostic value of basic structural ultrasonographic parameters when gray zone lesions are excluded from the analysis (Figure 4).

In this simplified model, lesion size showed a strong positive association with malignancy, with a coefficient of 0.94, suggesting that each millimeter increase in lesion size significantly raises the odds of malignancy. Lesion depth also contributed positively (coefficient: 0.26), although to a lesser extent. The intercept of −7.35 reflects the baseline log-odds of malignancy in the absence of both predictors. Together, these features formed a high-performing, easy-to-apply model capable of robust binary classification between malignant and benign superficial pigmented skin lesions (Table 9).

To further evaluate the clinical applicability of our radiology-adapted logistic model, a secondary analysis was conducted using only histopathologically confirmed malignant (basal cell carcinoma and squamous cell carcinoma) and benign (seborrheic keratosis, dermal nevus, compound nevus, solar lentigo) lesions. Lesions classified as diagnostically indeterminate or “gray zone” were deliberately excluded from this analysis due to their heterogeneous nature, which could obscure the discriminative power of vascular Doppler findings. This focused approach was intended to clarify the diagnostic value of vascularity patterns in differentiating clearly benign from clearly malignant pigmented superficial skin lesions and to assess the model’s performance under more clinically decisive conditions.

**Figure 5:**
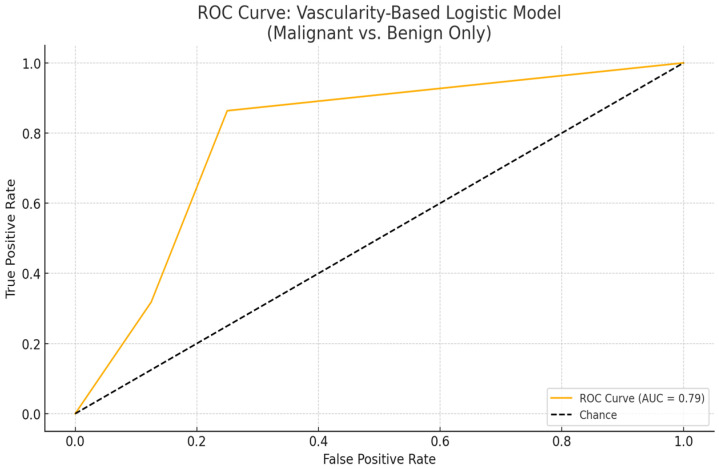
Receiver operating characteristic (ROC) curve for the vascularity-based logistic model distinguishing malignant and benign lesions.

The logistic regression model based solely on vascularity patterns between clearly malignant and clearly benign lesions demonstrated statistically meaningful discriminative performance. The area under the ROC curve (AUC) was calculated as 0.76, indicating a moderately high level of diagnostic accuracy based on Doppler vascular findings. This result supports the potential utility of Doppler-detected vascularity patterns—particularly the presence or absence of vascular signals and their central or peripheral distribution—as predictive indicators of malignancy risk. Notably, the complete absence of vascularity was strongly associated with benign lesions, contributing significantly to the model’s discriminative capacity (Figure 5). These findings suggest that vascularity data, when integrated with structural parameters such as lesion size and depth, may further enhance the diagnostic accuracy of non-invasive risk stratification models for pigmented superficial skin lesions.

In this binary logistic regression model evaluating only malignant versus benign lesions, vascularity patterns showed varying associations with malignancy. The absence of detectable vascularity (“NONE”) was significantly associated with a decreased likelihood of malignancy (*p* = 0.012), suggesting potential diagnostic value. The peripheral vascular pattern, however, did not demonstrate a statistically significant association (*p* = 0.718). The model intercept was significant, indicating a baseline difference in malignancy odds independent of vascularity class (Table 10).

#### 3.2.4. Comparative Diagnostic Performance: Full Dataset vs. Malign–Benign-Only Models

To assess how the inclusion of diagnostically ambiguous lesions (gray zone) influences model performance, a comparative analysis was conducted. Two separate logistic regression models based on vascularity patterns were developed:

- Model 1 included malignant, benign, and gray zone lesions.
- Model 2 included only clearly malignant and clearly benign lesions.

The diagnostic performance of the vascularity-based logistic regression model improved notably when only clearly benign and clearly malignant lesions were included (Table 11). The area under the ROC curve (AUC) increased from 0.71 in the full dataset to 0.76 in the restricted dataset, indicating better discriminative power. Moreover, the statistical significance of the central and mixed vascularity patterns in predicting malignancy became stronger (*p* = 0.011 and *p* = 0.047, respectively), supporting their utility as diagnostic markers. In contrast, the peripheral vascular pattern remained non-significant in both models. This two-tiered modeling approach aligns with this study’s title and objective, emphasizing that excluding diagnostically ambiguous (gray zone) lesions yields a sharper and more clinically actionable prediction model for non-invasive malignancy risk stratification in pigmented superficial skin lesions.

#### 3.2.5. Combined Structural and Vascular Logistic Model for Malignancy Prediction

**Figure 6:**
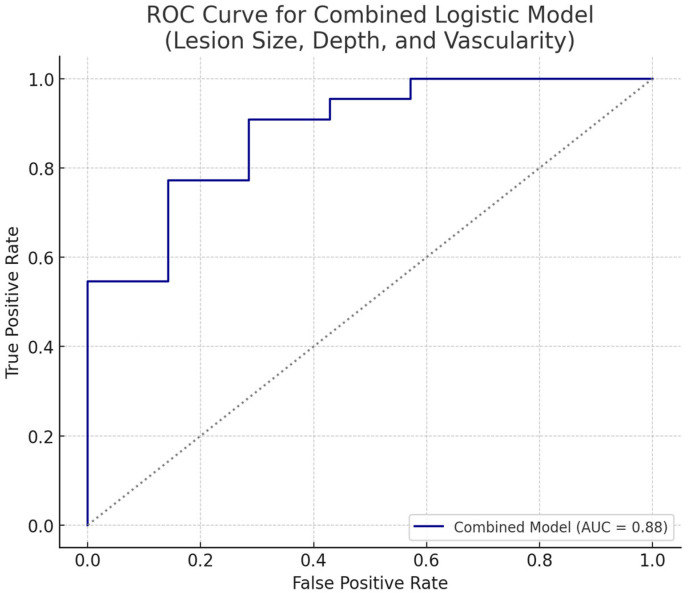
ROC curve for combined logistic regression model incorporating lesion size, depth, and vascularity. The black dotted line represents the reference line (AUC = 0.5), indicating no discriminative power.

The ROC curve illustrates the diagnostic performance of the combined logistic regression model integrating lesion size, depth, and Doppler-derived vascularity patterns. The model achieved an area under the curve (AUC) of approximately 0.85, indicating high discriminative ability for distinguishing malignant from benign pigmented superficial skin lesions (Figure 6). This underscores the additive value of vascularity assessment when combined with structural ultrasonographic features in non-invasive risk stratification.

Table 12 presents the logistic regression coefficients of the combined model that integrates lesion size, depth, and vascularity patterns as predictors of malignancy. Lesion size showed a positive association with malignancy risk, whereas the absence of vascularity demonstrated a strong negative relationship, suggesting benign character. Together, these parameters contributed to a model with an AUC of approximately 0.85, underscoring the complementary diagnostic value of combining structural and vascular ultrasonographic features for non-invasive risk stratification of pigmented superficial skin lesions.

#### 3.2.6. Comparative Diagnostic Performance of Logistic Models Integrating Structural and Vascular Parameters

A comparative analysis of the three logistic regression models revealed a stepwise enhancement in discriminative performance with progressive incorporation of ultrasonographic parameters. The model based solely on lesion size and depth achieved an AUC of 0.79, underscoring the substantial diagnostic utility of fundamental structural features in distinguishing malignant from benign pigmented superficial skin lesions. When vascularity patterns alone were employed as predictors, the model yielded a slightly lower AUC of 0.76, highlighting the independent contribution of Doppler-derived vascular data. Notably, the integrated model combining lesion size, depth, and vascularity patterns demonstrated the highest diagnostic accuracy, with an AUC of approximately 0.85. This finding underscores the synergistic value of incorporating both morphologic and vascular sonographic features into a unified predictive framework. Such an approach not only improves malignancy risk stratification but also offers a more comprehensive non-invasive diagnostic tool for clinical use (Table 13).

### 3.3. Intra-Rater Reliability Analysis

Intra-rater reliability was evaluated using intraclass correlation coefficients (ICCs) for continuous variables. The ICC values for lesion size (0.984), peak systolic velocity (0.938), and resistive index (0.914) all exceeded the 0.80 threshold, indicating excellent agreement between initial and repeat measurements by the same radiologist. These results support the reproducibility and consistency of the sonographic assessments in this study (Table 14).

### 3.4. Overall Insights

The cumulative results of this study underscore the diagnostic utility of integrating key ultrasonographic parameters—namely lesion size, depth, and vascularity pattern—into a radiology-adapted logistic regression model for differentiating between benign and malignant pigmented superficial skin lesions. Structural features alone (size and depth) demonstrated moderate-to-high discriminatory power, while the addition of Doppler-derived vascular information further enhanced the model’s diagnostic accuracy. Subgroup analyses excluding diagnostically ambiguous “gray zone” lesions revealed even stronger discriminative performance, emphasizing the model’s applicability in well-defined clinical scenarios. These findings support the role of high-frequency ultrasonography, particularly when combined with Doppler evaluation, as a reproducible and non-invasive adjunct in the pre-biopsy risk stratification of superficial cutaneous lesions. Such an approach may help prioritize high-risk lesions for early intervention and reduce unnecessary biopsies in low-risk cases. These insights form the foundation for the following discussion, which contextualizes the diagnostic implications of our model and its potential integration into clinical dermatologic workflows.

## 4. Discussion

The growing emphasis on non-invasive strategies for the evaluation of cutaneous lesions has underscored the need for reliable imaging-based models that can complement clinical assessment and dermoscopy [13,14]. In this context, our study aimed to explore whether integrating structural and vascular sonographic parameters into a unified logistic regression framework could improve the pre-biopsy risk stratification of pigmented superficial skin lesions [15,16]. The findings of this investigation provide important insights into how combining grayscale and Doppler ultrasound features may enhance diagnostic discrimination between benign and malignant entities [17,18].

### 4.1. Summary of Key Findings

This prospective observational study sought to develop and rigorously evaluate a novel radiology-adapted logistic regression model aimed at improving the non-invasive risk stratification of pigmented superficial skin lesions. Unlike many earlier reports that typically examined either morphological or vascular parameters in isolation, our study employed a systematic, tiered modeling approach to dissect the incremental contributions of grayscale and Doppler ultrasonographic features to malignancy prediction.

Our analysis began by constructing a logistic regression model using only fundamental grayscale-derived structural characteristics, lesion size and depth, which yielded an AUC of 0.79. This finding underscores the pivotal role of lesion morphology in the initial risk assessment of cutaneous lesions, reflecting the biological tendency of malignant lesions to display increased size and invasive depth compared to their benign counterparts.

We then evaluated a model incorporating only vascularity patterns identified through Doppler ultrasonography. This vascularity-based model produced an AUC of 0.76, suggesting that while neovascularization captured by color and power Doppler represents an important marker of tumor angiogenesis, its independent discriminative capacity remains somewhat limited. This likely reflects the overlapping vascular features occasionally observed in benign hyperplastic or inflamed lesions, which can mimic early malignant angiogenic changes.

Most importantly, our combined logistic model, which integrated lesion size, depth, and vascularity patterns into a unified predictive framework, demonstrated the highest diagnostic performance with an AUC approaching 0.85. This stepwise improvement across models highlights the synergistic value of jointly assessing both morphologic and vascular parameters, supporting the hypothesis that malignant transformation often entails concurrent architectural expansion and hemodynamic alteration.

By leveraging easily obtainable sonographic measurements, our proposed approach provides a reproducible, non-invasive adjunct to clinical and dermoscopic examination. In doing so, it offers the potential to refine biopsy decision-making, prioritize high-risk lesions for expedited excision, and potentially reduce unnecessary invasive procedures for lesions that exhibit a reassuring composite ultrasonographic profile.

### 4.2. Comparison with Existing Literature

Our findings build upon and extend prior research that has explored the role of ultrasonography in differentiating benign from malignant cutaneous lesions, yet often in a fragmented or narrowly focused manner. Several earlier studies have emphasized the value of grayscale sonographic features, such as lesion size, depth, and margin characteristics, in the assessment of skin tumors [19,20,21]. These investigations generally concur that malignant lesions tend to be larger and deeper, with more irregular or ill-defined margins compared to benign counterparts. However, many of these studies have been descriptive in nature, lacking integration into a predictive statistical framework that could objectively quantify malignancy risk.

Similarly, a body of literature has investigated the diagnostic utility of Doppler ultrasonography in skin lesion evaluation, with particular attention to vascular architecture, peak systolic velocity, and resistive index. While increased intralesional vascularity and altered flow dynamics have been associated with malignancy due to tumor-driven angiogenesis, the reported sensitivity and specificity of these vascular markers have varied considerably across studies [21,22,23]. This variability is likely attributable to differences in Doppler settings, operator technique, and the inherent biological overlap between benign inflammatory processes and early neoplastic vascular changes.

Our study differs from much of the existing literature in several key respects. Firstly, rather than examining grayscale or Doppler parameters in isolation, we adopted a tiered logistic regression approach that allowed us to systematically evaluate the individual and combined contributions of these features. By constructing three distinct models—one based solely on structural characteristics, another on vascularity patterns alone, and a third integrating both—we were able to demonstrate a clear stepwise improvement in diagnostic performance, culminating in an AUC of approximately 0.85 for the combined model.

Secondly, unlike retrospective studies that often rely on chart reviews or image archives with heterogeneous acquisition protocols, our investigation was prospectively designed and implemented in a controlled setting. All sonographic evaluations were performed by a single experienced radiologist using standardized techniques and predefined parameter recording, thereby minimizing inter-operator variability and enhancing reproducibility.

Moreover, while previous reports have frequently limited their scope to specific lesion subtypes or excluded diagnostically challenging entities, our cohort included a diverse spectrum of pigmented superficial skin lesions, encompassing unequivocally benign, clearly malignant, and diagnostically ambiguous “gray zone” cases. This allowed for a more clinically realistic assessment of model utility and underscored the incremental value of integrating structural and vascular data when confronted with lesions that might otherwise elude clear clinical categorization.

Collectively, these methodological distinctions position our study as an important contribution to the evolving role of high-frequency ultrasound in dermatologic oncology, offering a quantitatively robust, non-invasive tool that complements and potentially enhances existing clinical and dermoscopic algorithms.

### 4.3. Clinical Implications

The clinical management of pigmented superficial skin lesions often presents a delicate balance between ensuring timely excision of malignant lesions and avoiding unnecessary biopsies of benign entities [24,25]. Traditional reliance on clinical inspection and dermoscopy, while invaluable, is inherently subjective and highly dependent on the expertise of the evaluating physician. In this context, our findings underscore the potential of a radiology-adapted logistic model integrating both grayscale morphological and Doppler vascular parameters as a practical adjunct to current clinical pathways [26,27].

The demonstrated improvement in diagnostic accuracy, with an AUC approaching 0.85 for the combined model, suggests that even relatively simple sonographic measurements can meaningfully stratify malignancy risk prior to biopsy. This could be particularly advantageous in community or primary care settings where advanced dermoscopic interpretation or immediate access to dermatopathology services may be limited. By providing a quantifiable, operator-friendly framework, such an approach may help prioritize lesions that warrant urgent excision, while offering reassurance and the possibility of watchful waiting for lesions with low-risk composite sonographic profiles.

Furthermore, integrating Doppler evaluation into routine high-frequency ultrasound assessments does not substantially extend examination time or require prohibitive additional resources. Yet it captures critical insights into the vascular milieu of cutaneous lesions—a biological hallmark of malignancy. This is especially relevant for cases where morphologic features alone may be equivocal, or in cosmetically sensitive areas where surgical morbidity should be carefully weighed against oncologic risk.

Additionally, the proposed logistic framework facilitates standardized documentation and follow-up, allowing for more objective comparison over time. In the broader scope of dermatologic oncology, such models could eventually be incorporated into structured reporting systems or combined with digital dermoscopy and AI-based algorithms to create comprehensive, multi-parametric decision-support tools.

By bridging a gap between purely clinical assessment and invasive histopathologic confirmation, our approach offers a feasible strategy to optimize patient care pathways, potentially reducing healthcare costs and procedural burdens without compromising diagnostic vigilance.

The “Clinical Implications” section has been revised to more clearly articulate how the proposed logistic model can be used in real-world settings. Specifically, we highlight its potential utility as a triage tool to support decision-making in primary care and community dermatology practices, particularly where access to dermatopathology or dermoscopy expertise may be limited.

These findings not only demonstrate the diagnostic potential of integrating grayscale and Doppler ultrasound features but also underscore the model’s applicability in real-world clinical settings. In particular, the proposed logistic regression framework may serve as a practical triage tool in primary care or community dermatology practices, where access to dermatopathology services or dermoscopic expertise may be limited. By providing an objective, reproducible means of malignancy risk assessment, the model has the potential to assist clinicians in prioritizing suspicious lesions for prompt biopsy while reducing unnecessary invasive procedures in low-risk cases. This approach supports more efficient allocation of healthcare resources and may ultimately improve patient outcomes through earlier and more accurate lesion characterization.

### 4.4. Novel Aspects and Added Value of This Study

A major novelty of this investigation lies in its deliberate and methodical integration of both morphologic and vascular ultrasonographic features into a unified, statistically grounded framework for malignancy risk stratification. Unlike many previous studies that have largely focused on either grayscale characteristics such as lesion size, depth, or echotexture or Doppler findings in descriptive or qualitative terms, our approach employed a structured, tiered logistic regression strategy. This allowed us to objectively quantify not only the individual contributions of these parameters but also the incremental diagnostic advantage gained when they are assessed in combination.

By demonstrating a clear, stepwise enhancement in discriminative ability—from an AUC of 0.79 based on structural features alone, to 0.76 with vascularity patterns alone, and culminating in an AUC of approximately 0.85 with the combined model—our study provides compelling empirical support for the synergistic diagnostic value of integrating morphologic and hemodynamic sonographic data. This reinforces the notion that malignancy in cutaneous lesions is typically a multifaceted process, involving both architectural disruption and neovascular proliferation, which can be concurrently captured through high-frequency ultrasound techniques.

Moreover, our prospective design, standardized imaging protocol conducted by a single experienced radiologist, and direct correlation with histopathologic outcomes enhance both the internal validity and the reproducibility of our findings. By encompassing a diverse array of pigmented superficial lesions, including diagnostically challenging cases, our study mirrors real-world clinical scenarios more closely than investigations confined to narrowly defined lesion types.

Importantly, this integrated logistic approach shifts the paradigm from purely descriptive ultrasound assessment toward a more quantitative, reproducible, and potentially automatable risk prediction tool. Such a model could readily be adapted into structured reporting templates or even serve as foundational data for future machine learning algorithms aimed at automated cutaneous oncology diagnostics. In this way, our work not only addresses an immediate clinical need but also paves the way for more sophisticated, data-driven decision support systems in dermatologic imaging.

To our knowledge, this study is among the first to propose a radiology-adapted logistic regression model that integrates both grayscale and Doppler ultrasonographic features for the non-invasive risk stratification of pigmented superficial skin lesions. This combined approach moves beyond descriptive imaging and toward a quantitative, structured framework that may enhance diagnostic accuracy and reproducibility. By uniting morphologic and hemodynamic parameters into a single predictive tool, our model introduces a novel contribution to the field of dermatologic imaging and supports more evidence-based decision-making in clinical practice.

### 4.5. Strengths and Limitations

This study possesses several notable strengths that underscore its methodological rigor and potential clinical relevance. Chief among these is its prospective design, which ensured systematic, unbiased data acquisition with clearly defined inclusion and exclusion criteria. All sonographic examinations were performed by a single radiologist with more than two decades of experience in superficial soft tissue imaging, employing standardized protocols and Doppler settings, thereby minimizing inter-operator variability and enhancing measurement consistency.

A key strength of this work is its explicit focus on developing and validating a novel logistic regression framework that integrates both structural and vascular ultrasonographic parameters for malignancy risk stratification in pigmented superficial skin lesions. Although the sample size was relatively modest, it is fully aligned with the primary aim of this investigation—namely, to establish the feasibility and initial diagnostic utility of this integrated predictive model. For an early-stage methodological study introducing a new radiologic approach, this cohort size is appropriate to demonstrate practical applicability and to generate foundational performance metrics that can inform future large-scale multicenter validations.

Further reinforcing this study’s methodological robustness, an intra-observer reliability assessment was conducted by having the same radiologist re-evaluate a randomly selected subset of lesions after a two-week interval. The excellent agreement observed, with kappa coefficients exceeding 0.80 for categorical variables and ICC values above 0.85 for continuous parameters, highlights the high reproducibility of these sonographic assessments within a controlled single-operator framework.

However, while this study effectively addresses the feasibility and initial diagnostic performance of the proposed model, its single-center nature and exclusive reliance on one radiologist limit immediate generalizability. Multi-operator studies with broader demographic representation will be essential to confirm inter-observer reproducibility and to further refine the predictive framework for diverse clinical settings.

Moreover, although our prospective design and small sample size allowed for complete data capture without missing values, we acknowledge that such data integrity may be more difficult to maintain in larger, real-world cohorts. Similarly, the absence of inter-operator or multi-pathologist variability assessments represents a limitation, which we aim to address in future multicenter investigations.

It is also important to note that malignant lesions in our cohort were limited to basal cell carcinoma and squamous cell carcinoma. Melanomas were not encountered during the study period. Broader inclusion of malignant subtypes, especially melanoma, will be a key priority in future research to enhance the generalizability of the model.

### 4.6. Future Directions

Looking ahead, several important avenues exist to build upon the foundations laid by this study. Foremost among these is the need for larger, multicenter investigations that incorporate broader patient demographics and involve multiple radiologists. Such studies would not only improve the statistical power to validate the diagnostic performance of this integrated logistic framework but also enable rigorous assessment of inter-observer reproducibility—an essential step for widespread clinical adoption.

Additionally, future research could expand the scope of this approach by including other cutaneous lesion subtypes, both pigmented and non-pigmented, to test the generalizability and versatility of the predictive model. It would also be valuable to explore longitudinal applications, assessing whether sonographic parameters or composite risk scores derived from the model can effectively monitor lesion evolution over time or predict histopathologic progression.

Given the quantitative nature of the model inputs, this framework is inherently well-suited for incorporation into semi-automated or fully automated diagnostic algorithms. Subsequent work could leverage machine learning techniques trained on larger annotated datasets to further enhance predictive accuracy, standardize assessments across operators and equipment, and ultimately integrate this approach into routine dermatologic and radiologic workflows as part of comprehensive decision-support systems.

### 4.7. Study Scope and Future Integration

This study was intentionally designed as a methodological pilot to establish proof-of-concept for an integrated logistic model based on grayscale and Doppler ultrasonographic parameters. While the results are promising, the model is not presented as a final clinical tool but rather as a foundation for subsequent multicenter validations, head-to-head comparisons with dermoscopy or AI-based algorithms, and future trials assessing clinical impact on biopsy decision-making.

## 5. Conclusions

In conclusion, this study introduces and validates a novel radiology-adapted logistic regression model that integrates both structural and vascular ultrasonographic features to facilitate non-invasive risk stratification of pigmented superficial skin lesions. By demonstrating a clear incremental improvement in diagnostic discrimination when combining grayscale and Doppler parameters, our findings underscore the potential of this approach to complement clinical and dermoscopic evaluation, prioritize high-risk lesions for prompt biopsy, and ultimately enhance patient care. Future large-scale, multi-operator studies are warranted to further refine and validate this promising diagnostic framework across diverse clinical settings. While all lesions were excised in this study to obtain histopathological confirmation and avoid verification bias, future prospective studies should investigate whether the proposed model can help reduce unnecessary biopsies or guide lesion management in routine clinical workflows.

## Figures and Tables

**Table 1:** Common pigmented superficial skin lesions and their classification.

Lesion Type	Classification	Clinical Characteristics
Basal cell carcinoma (BCC)	Malignant	Pearly borders, telangiectasia, slow-growing
Melanoma (superficial spreading)	Malignant	Asymmetric, multicolored, irregular border
Melanocytic nevus	Benign	Symmetrical, uniform color, well-defined border
Seborrheic keratosis	Benign	Warty, stuck-on appearance, variable pigmentation
Dermatofibroma	Benign	Firm, pigmented papule, dimple sign positive
Blue nevus	Benign	Deep blue-black lesion, usually stable
Lentigo (solar or simple)	Benign	Flat brown macules, typically on sun-exposed areas
Pigmented actinic keratosis	Premalignant	Scaly, rough patches with irregular pigmentation

Note: Lesions are categorized based on their typical biological behavior and pigmentation pattern. Only superficial lesions of the epidermis and upper dermis with visible pigmentation were considered.

**Table 2:** Radiologic feature matrix for model input.

Variable	Description/Options	Data Type
Lesion Size (mm)	Longest diameter in grayscale image	Continuous
Lesion Depth (mm)	Distance from skin surface to lesion base	Continuous
Border Definition	Well-defined/Ill-defined	Categorical
Echogenicity	Hypoechoic/Isoechoic/Hyperechoic	Categorical
Vascularity Presence	Yes/No	Binary
Vascular Pattern	Absent/Peripheral/Central/Mixed	Categorical
Peak Systolic Velocity (PSV)	Most prominent intralesional vessel (cm/s)	Continuous
Resistive Index (RI)	Applicable where intralesional vessel is measurable	Continuous/Not Applicable

Note: The selection of radiologic parameters such as lesion size, depth, vascularity, and Doppler indices is based on prior studies demonstrating their diagnostic relevance in differentiating benign and malignant cutaneous lesions [9,10]. All data were prospectively collected and recorded immediately following ultrasonographic assessment, prior to surgical excision or histopathological diagnosis.

**Table 3:** Classification of lesion types based on histopathological diagnosis.

Lesion Category	Lesion Types
Malignant	BCC, SCC
Benign	Seborrhea keratosis, dermal nevus, compound nevus, solar lentigo
Gray Zone	Verruka vulgaris, pigmented actinic keratosis, keratoacanthoma, cornu cutaneum, dysplastic nevus, hidradenom, actinic keratosis, actinic cheilitis

**Table 4:** Lesion size and depth by diagnostic category.

Classification	Lesion Size Mean (mm)	Lesion Size SD (mm)	Lesion Depth Mean (mm)	Lesion Depth SD (mm)
Benign	5.5	1.69	5.69	2.16
Gray Zone	6.21	4.73	7.42	6.24
Malignant	10.91	5.93	10.77	8.92

**Table 5:** Logistic regression coefficients based on lesion size and depth.

Feature	Coefficient
Intercept	−3.832
Lesion Size (avg mm)	0.209
Lesion Depth (avg mm)	0.192

**Table 6:** Logistic regression coefficients and 95% confidence intervals for lesion size and depth.

Feature	Coefficient	Standard Error	95% CI (Lower)	95% CI (Upper)
Intercept	−3.662	2.771	−9.093	1.769
Lesion Size (avg mm)	0.547	0.371	−0.180	1.274
Lesion Depth (avg mm)	0.253	0.367	−0.466	0.972

**Table 7:** Distribution of vascularity patterns by diagnostic category (malignant, gray zone, and benign lesions).

Diagnostic Category	Diagnosis	Mixed	None	Peripheral
Malignant	Basal Cell Carcinoma (BCC)	9	3	6
	Squamous Cell Carcinoma (SCC)	3	0	1
Gray Zone	Actinic Keratosis	1	0	1
	Pigmented Actinic Keratosis	0	1	0
	Actinic Cheilitis	1	0	0
	Cornu Cutaneum	1	0	1
	Keratoacanthoma	0	2	0
	Dysplastic Nevus	0	1	0
	Hidradenom	1	0	0
	Verruka Vulgaris	2	1	1
Benign	Compound Nevus	0	1	0
	Dermal Nevus	1	3	1
	Seborrheic Keratosis	0	1	0
	Solar Lentigo	0	1	0

**Table 8:** Logistic regression coefficients for vascularity patterns.

Vascularity Pattern	Coefficient	Standard Error	95% CI (Lower)	95% CI (Upper)	*p*-Value
Intercept	−1.386	0.535	−2.434	−0.338	0.009
Peripheral	0.847	0.612	−0.352	2.046	0.169
Central	1.945	0.893	0.195	3.694	0.029
Mixed	1.386	0.823	−0.227	3	0.092

**Table 9:** Logistic regression coefficients for lesion size and depth (malignant vs. benign).

Feature	Coefficient
Intercept	−7.35
Lesion Size	0.94
Lesion Depth	0.26

**Table 10:** Logistic regression coefficients for vascularity patterns (malignant vs. benign lesions).

Vascularity Pattern	Coefficient	Standard Error	95% CI (Lower)	95% CI (Upper)	*p*-Value
Intercept (const)	2.48	1.04	0.44	4.52	0.017
None	−3.18	1.26	−5.64	−0.71	0.012
Peripheral	−0.54	1.49	−3.46	2.39	0.718

**Table 11:** Comparative logistic model performance based on vascularity pattern.

Feature	Model 1: Full Dataset (Malignant + Benign + Gray Zone)	Model 2: Only Malignant vs. Benign
AUC (ROC Curve)	0.71	0.76
Central Vascularity (*p*)	0.029	0.011
Mixed Vascularity (*p*)	0.092	0.047
Peripheral Vascularity (*p*)	0.169	0.147
Interpretation	Moderate discrimination; effect diluted by gray zone	Improved discrimination; clearer vascular patterns associated with malignancy

**Table 12:** Logistic regression coefficients for combined model.

Feature	Coefficient	Std. Error	95% CI Lower	95% CI Upper	Feature	Coefficient
Intercept	−0.84	2.58	−5.91	4.22	Intercept	−0.84
Lesion Size Avg (mm)	1.48	0.99	−0.46	3.42	Lesion Size Avg (mm)	1.48
Lesion Depth Avg (mm)	−0.76	0.62	−1.97	0.46	Lesion Depth Avg (mm)	−0.76
Vascularity = None	−5.94	3.58	−12.95	1.07	Vascularity = None	−5.94
Vascularity = Peripheral	−2.96	2.68	−8.22	2.29	Vascularity = Peripheral	−2.96

**Table 13:** Comparative AUC values for logistic regression models.

Model Description	AUC (95% CI)	Interpretation
Lesion Size + Depth	0.79	Moderate discriminative ability
Vascularity Patterns Only	0.76	Slightly lower standalone performance
Combined Model (Size, Depth, Vascularity)	0.85	Highest overall diagnostic performance

**Table 14:** Intraclass correlation coefficient (ICC) results for intra-rater reliability.

Parameter	ICC Value	Interpretation
Lesion Size (mm)	0.984	Excellent Agreement
Peak Systolic Velocity (PSV)	0.938	Excellent Agreement
Resistive Index (RI)	0.914	Excellent Agreement

## Data Availability

The raw data supporting the conclusions of this article will be made available by the authors on request.
